# Analysis of smart imaging runtime

**DOI:** 10.1186/s42649-025-00115-5

**Published:** 2025-08-14

**Authors:** Thomas Athey, Shashata Sawmya, Yaron Meirovitch, Richard Schalek, Pavel Potocek, Ishaan Chandok, Maurice Peemen, Jeff Lichtman, Aravinthan Samuel, Nir Shavit

**Affiliations:** 1 Massachusetts Institute of Technology, Cambridge, MA USA; 2 Harvard University, Cambridge, MA USA; 3 Thermo Fisher Scientific, Eindhoven, the Netherlands; 4 Saarland University, Saarbrücken, Germany; 5 Red Hat, Inc., Boston, MA USA

**Keywords:** Electron microscopy, Deep learning, Parallelization, Mixed-precision, Active acquisition, Runtime

## Abstract

Smart microscopy is a new imaging approach that involves rapid imaging, prediction of important subregions, then selective re-imaging. This approach has been validated in reducing imaging beam time in electron microscopy connectomics, but the speedup depends on various imaging workflow parameters. Here we present the first runtime analysis of traditional vs. smart microscopy and show how these parameters can magnify, or diminish potential time savings. We provide a GUI application that calculates the theoretical time savings of smart microscopy from user input parameters describing their imaging workflow. Finally, we measure end-to-end runtime of SmartEM acquisition on an electron microscope to demonstrate two strategies for faster acquisition: mixed-precision neural networks and parallelization of microscope and support computer operations.

## Introduction

**Fig. 1:**
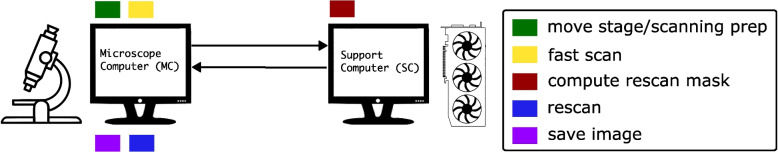
SmartEM acquisition involves microscope acquisition, and machine learning inference. In this implementation, a microscope computer executes stage movements and scanning preparation (e.g. auto-focusing), followed by a fast scan. The fast scan is sent to a support computer which uses neural network inference to identify which areas should be rescanned. The microscope computer then executes rescanning and saves the result, which is a combination of the fast scan and the rescan

The framework of smart microscopy was inspired by the observation that information content (with respect to some imaging goal) may be distributed inhomogeneously across biological specimens. In connectomics, for example, it may be more important to image synapses than blood vessels or glial cells. Smart microscopy balances the tradeoff between signal-to-noise ratio and imaging speed by allocating acquisition time according to objects of interest. First, there is a “fast” acquisition which gives an overview of the imaging sample. Then, an algorithm uses the fast image to generate a *rescan mask*, predicting which regions need to be imaged in greater detail. Finally, a “slow” acquisition step selectively images those regions (Anderson et al. 2013; Meirovitch et al. 2024; Mi et al. 2020; Potocek et al. 2020) (Fig. 1).

**Fig. 2:**
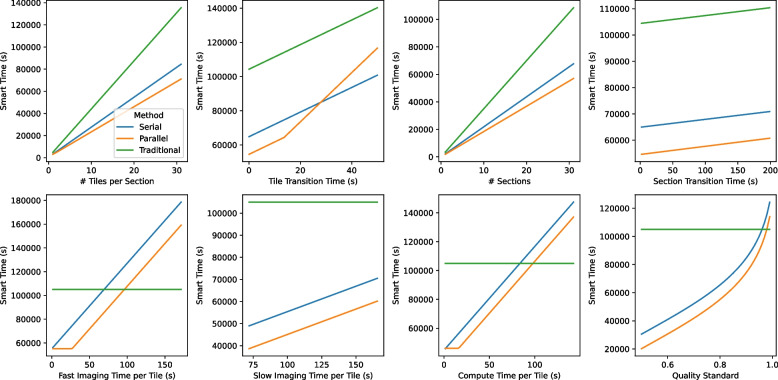
Image acquisition parameters affect how much time is saved using smart microscopy. In general, larger imaging batches (increased number of sections or tiles per section) lead to increased time savings, but slower imaging rates, slower computation rates, or higher quality standards reduce the time savings of smart microscopy. In each plot, one parameter is varied while the others are kept constant at their default values from Table 1

**Fig. 3:**
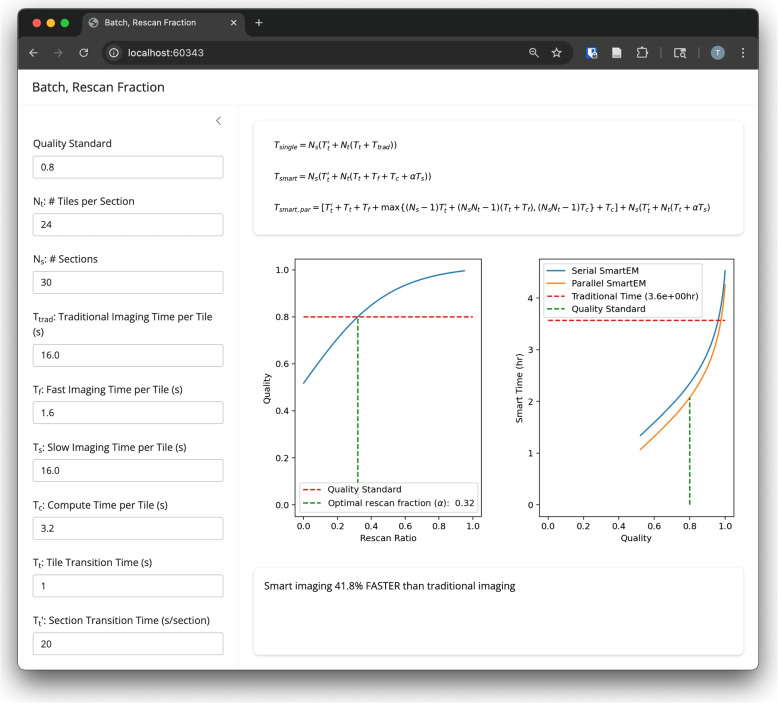
Our Shiny (The Shiny development team n.a.) Python app allows users to enter the parameters of their acquisition workflow, then visualize how much acquisition time could be saved using the serial or parallel versions of SmartEM. The left panel contains fields for user-specified parameters. The top-right shows the runtime equations in our model. The center-right shows plots of quality vs. rescan ratio and runtime vs. quality with vertical lines at the operating point that achieves the quality standard. The bottom-right panel states whether traditional or smart microscopy is faster, and by how much

Previous work in smart microscopy has focused on reducing imaging beam time in connectomics applications (Meirovitch et al. 2024), but in this work, we focus on the end-to-end time of the complete pipeline. We construct a model of the complete SmartEM workflow, which includes nine parameters (Table 1). We compare a smart imaging pipeline (fast imaging, with slow rescanning) to a traditional imaging pipeline (fixed imaging rate, no rescanning). We perform a sensitivity analysis on all parameters (except traditional imaging time per tile $T_{trad}$) and show that the theoretical time savings of smart microscopy varies drastically across different parameter values (Fig. 2). We present a GUI app that compares the runtimes of traditional and smart imaging under user-specified parameter values (Fig. 3). Finally, we present results of end-to-end timing experiments to demonstrate mixed-precision inference for faster computation, and parallelization of microscope and computation operations.

**Table 1:** Parameters for traditional and smart microscopy workflows in serial section imaging

Variable description	Symbol	Units	Default value
Quality Standard	Q	variable	0.8
Number of tiles per section	$N_t$	unitless	24
Number of sections	$N_s$	unitless	30
Traditional imaging time per tile	$T_{trad}$	s	144
Fast imaging time per tile	$T_f$	s	14.4
Slow imaging time per tile	$T_s$	s	144
Compute time per tile	$T_c$	s	28.8
Tile transition time	$T_t$	s	4
Section transition time	$T_t'$	s	20

## Main

The goal of a microscopy workflow is to produce images of adequate quality. The notion of quality depends on the experiment and could mean imaging coverage of a given tissue, or generation of images that can be accurately segmented for cells. In our model, we represent quality as a continuous quantity in the range [0, 1]. The Quality Standard *Q* is the quality level that needs to be achieved by the workflow.

In serial section microscopy, a tissue sample is sliced into $N_s$ sections and each section is imaged using $N_t$ fields of view (or *tiles*). There are overhead time costs in transitioning between tiles within a section ($T_t$), and between sections ($T_t'$).

In traditional imaging, it takes $T_{trad}$ time to image a single tile. In smart microscopy, the fast acquisition takes $T_f$ time per tile, and the slow acquisition takes $T_s$ time per tile. Additionally, it takes $T_c$ time for the smart microscopy system to compute the rescan mask for a single tile.

These variables are summarized in Table 1 and we computed default values for a hypothetical scattering electron microscopy (EM) workflow with $12,000 \times 12,000$ pixel tiles (Meirovitch et al. 2024; Kasthuri et al. 2015). For example, the default fast and slow imaging times are derived from multiplying the number of pixels by 100*ns*/*px* and 1000*ns*/*px* dwell times, respectively. The compute time was extrapolated from the time it took to run a U-Net on a $6144 \times 4096$ pixel image using a NVIDIA GeForce RTC 3090 Ti GPU - about 2.5 seconds. We accounted for the fact that the current SmartEM method involves two U-Nets in series, then used linear extrapolation to account for a larger tile size (Meirovitch et al. 2024). Tile transition times ($T_{t}$) account for stage movements on the order of $10-100$ microns ($\sim 1$s in our setting), plus some time for auto-functions such as auto-focusing. Transitions between sections take longer as they require larger stage movements, and more auto-functions to accommodate the topography of the new section (Goldstein et al. 2018).

Traditional image acquisition involves imaging at a fixed rate, and transitioning between fields of view. The overall imaging time for a traditional workflow is:

1 $$\begin{aligned} T_{single}=N_s(T_t'+N_t(T_t+T_{trad})) \end{aligned}$$

Here, we consider one free variable, the fraction of the image that was rescanned during slow acquisition, $\alpha$. Smart microscopy with $\alpha =0$ corresponds to traditional microscopy with $T_{trad}=T_f$ i.e. the entire sample is imaged exclusively at the fast acquisition rate. If fast acquisition, computational prediction, and slow acquisition are performed serially, then the end-to-end runtime for the smart workflow is:

2 $$\begin{aligned} T_{smart}=N_s(T_t'+N_t(T_t+T_f+T_c+\alpha T_s)) \end{aligned}$$

The $\alpha T_s$ term comes from the assumption that the time to image a subregion of the image is directly proportional to the size of the subregion.

It is possible to accelerate smart microscopy by performing region prediction and imaging simultaneously. In this setup, the first tile during fast acquisition is passed through the predictor while the next tile is imaged. Fast acquisition proceeds in this way, with region prediction happening in parallel. After fast acquisition and prediction covers all tiles, slow acquisition occurs. The end-to-end runtime for this workflow is:

3 $$\begin{aligned} T_{smart,par} & =( T_t' + T_t+T_f \nonumber \\ & \quad +\max \{(N_s-1)T_t'+(N_sN_t-1)(T_t+T_f), \nonumber \\ & \quad (N_sN_t-1)T_c\}+T_c) \nonumber \\ & \quad +N_s(T_t'+N_t(T_t+\alpha T_s)) \end{aligned}$$

We suggest choosing $\alpha$ in the following way. First, assume we have some known continuous quality function *q*(*x*) which maps rescan fractions to image quality values. Then, establish a quality standard such that $q(0)\le Q\le q(1)$. The lowest $\alpha$ that satisfies the quality standard can then be computed from *q*(*x*). In our experiments, we use the quality function

4 $$\begin{aligned} q(x) = \frac{1}{1+e^{-4x}}+\frac{e^{-4}}{1+e^{-4}} \end{aligned}$$

where we note that *q* increases monotonically from *q*(0) to 1. *q*(0) represents the image quality from fast imaging alone (no rescanning), so it is some value between 0 and 1. In our case, $q(0)=0.5$. In reality, the function *q* would need to be estimated for the task at hand.

We computed the traditional and smart microscopy runtimes over various values of the model parameters. When a parameter was not being varied, it was held constant at the default value from Table 1. The results in Fig. 2 show that parallel smart microscopy is often the fastest acquisition method. However, under some parameter values, traditional or serial smart microscopy might be the fastest method. In particular, the time savings from smart microscopy increase as the number of sections or tiles increase. On the other hand, smart microscopy becomes less desirable if tile transition time, fast or slow imaging time, computation time, or quality standard increases. Finally, section transition time seems to affect traditional and smart microscopy runtimes in the same way.

In order to allow for more comprehensive runtime analysis, we built a Shiny (The Shiny development team n.a.) based Python app, which can calculate and compare traditional and smart microscopy runtimes for user specified parameter values. Figure 3 shows a screenshot of the app, showing the ideal rescan fraction for a given quality standard, and whether this rescan fraction leads to time savings over traditional microscopy.

**Fig. 4:**
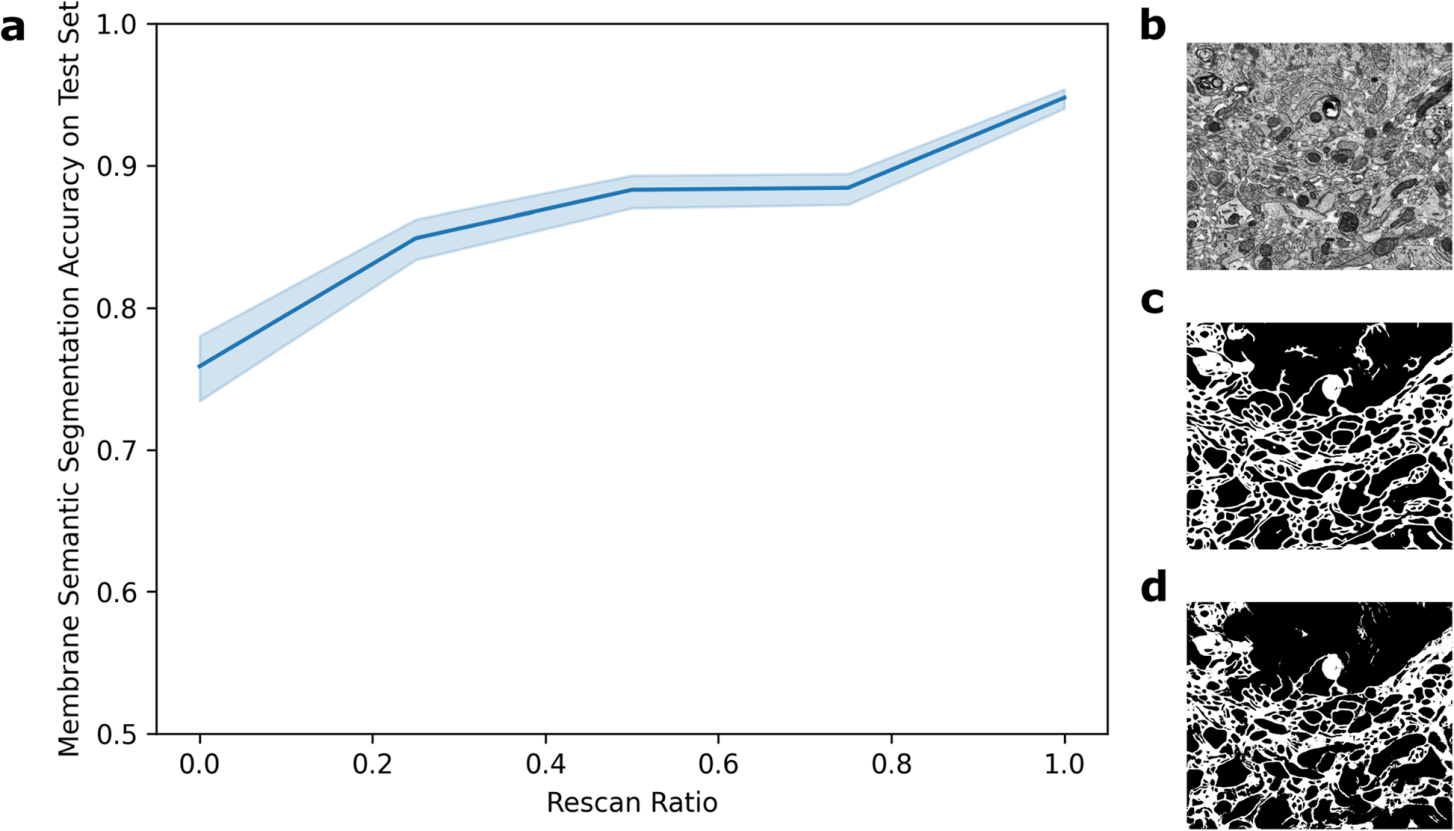
To demonstrate an example of estimating a quality function *q*, a collection of images was acquired at both 25 ns/pixel and 1200 ns/pixel then combined to produce mixed dwell time images. **a** An example quality function was estimated by evaluating a U-Net in cell membrane semantic segmentation at a range of rescan ratios. Shown are the mean and 95% confidence intervals of pixelwise accuracies across a held-out test set. An example of a mixed dwell time image is shown (**b**), with the corresponding ground truth (**c**) and U-Net predicted (**d**) membrane segmentations

Next, we demonstrated an approach to obtaining a quality function *q* on a dataset of 1640x1920 pixel EM images acquired at multiple dwell times, with accompanying ground-truth cell membrane semantic segmentations. In this example, the objective was membrane segmentation, so the quality function was defined to be membrane segmentation accuracy by a neural network. Mixed dwell time images were generated by combining 25 ns/pixel and 1200 ns/pixel images from the same field of view. In this context, the “rescan rate” corresponds to the fraction of the image that came from 1200 ns/pixel data. We trained a U-Net to perform binary segmentation on mixed dwell time images using a training set of 230 images and a validation set of 8 images. We evaluated the U-Net on a test set of 18 images at varying rescan rates and computed pixelwise binary segmentation accuracy (Fig. 4). U-Net architectures were from the Pytorch Connectomics package (Lin et al. 2021), and were trained for 20 epochs with an Adam optimizer (Kingma and Ba 2017).

Finally, we measured end-to-end runtime while acquiring three datasets, each under four acquisition settings on a Thermo Fisher Verios system. The first setting was traditional imaging, at a dwell time of 1000 ns/pixel. We then performed SmartEM using fast and slow scan dwell times of 100 ns/pixel and 800 ns/pixel respectively, with a rescan rate of 10%. In full-precision/serial mode, the microscope and support computer operations were performed serially, and rescan masks were computed using U-Nets from the Pytorch Connectomics package (Lin et al. 2021) with full floating point precision. In mixed-precision/serial mode, U-Nets with identical architectures were trained and executed using automatic mixed-precision (Paszke et al. 2019). The final setting included both mixed-precision inference, and parallelization of microscope and support computer operations. Parallelization involves performing all fast scans for a given section, and computing rescan masks in parallel, followed by rescanning all tiles for that section (Algs. 1 and 2). Parallelization was achieved by using multi-consumer queues from Python's queue package. The support computer that executed the neural networks was equipped with a NVIDIA GeForce RTC 3090 Ti GPU. The pixel resolution in all cases was 4 nm.

The first dataset was composed of a 6x6 tile grid across two tissue setions of mouse cortex (“Sample 1”), where each tile was 6144x4096 pixels. The second and third datasets were from a different sample of mouse cortex (“Sample 2”). The second dataset involved a 10x10 tile grid from a single section at 2048x1768 pixels per tile. The third dataset involved 6x6 tile grids across eight sections at 6144x4096 pixels per tile.

**Figure:**
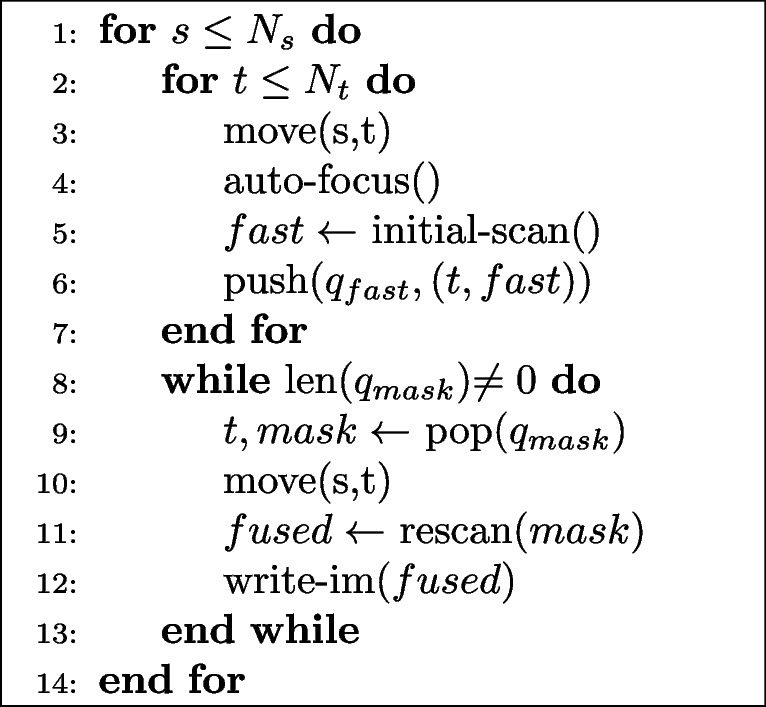
**Algorithm 1** Parallel SmartEM: imaging thread

**Figure:**
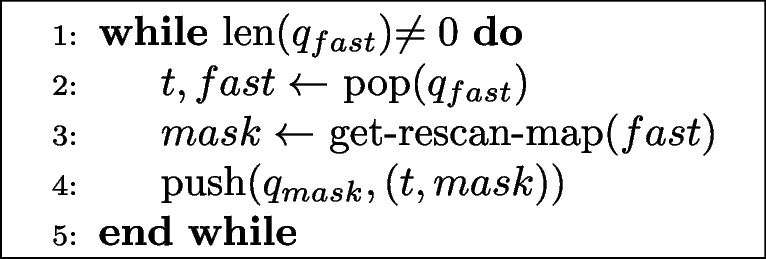
**Algorithm 2** Parallel SmartEM: computation thread

**Fig. 5:**
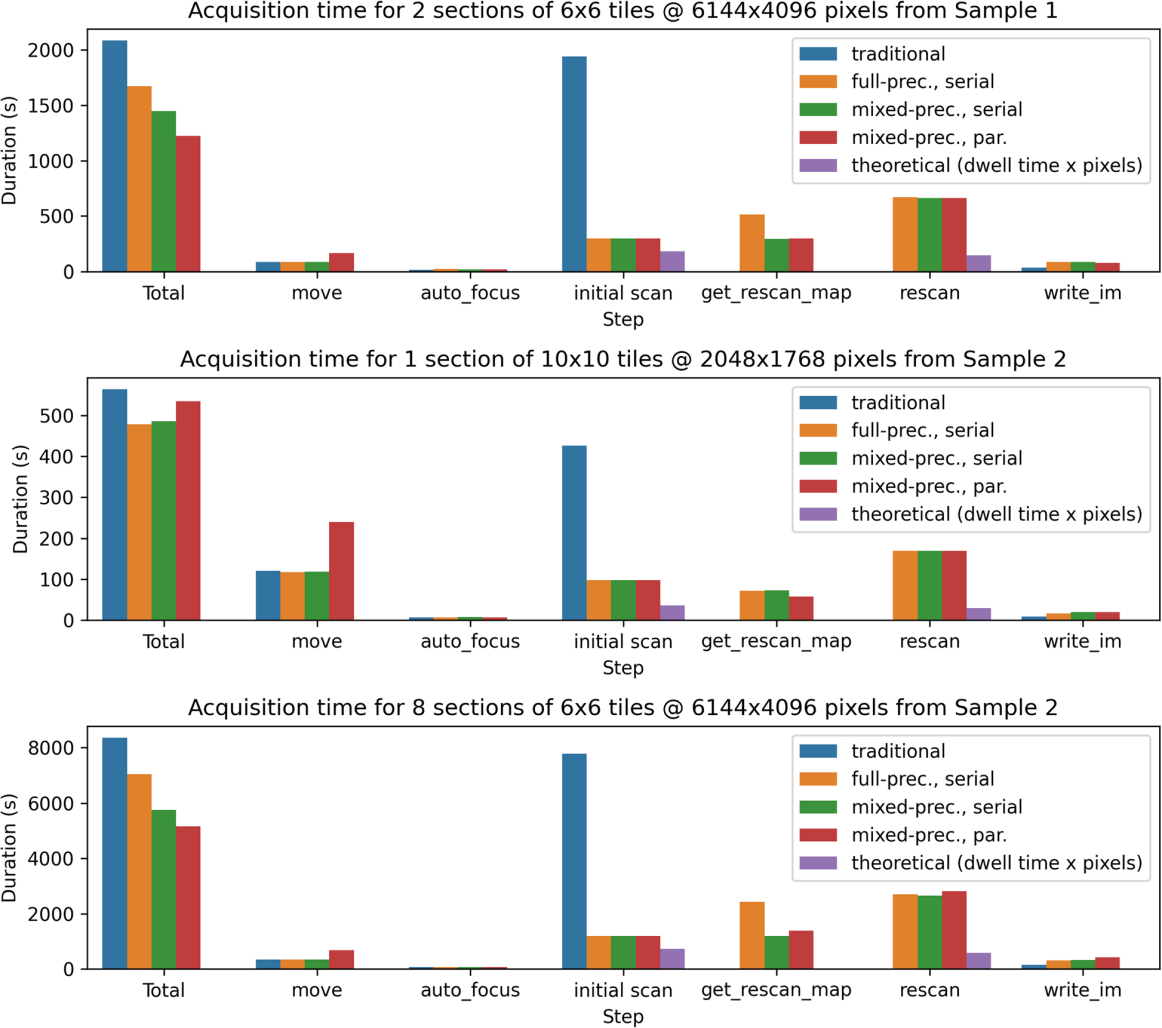
The end-to-end SmartEM acquisition time was measured under three settings: serial mode with full-precision neural networks (“full-prec., serial”), serial mode with autocasting for mixed-precision inference (“mixed-prec., serial”), and parallel mode with autocasting (“mixed-prec., par.”). The left set of bars show the total time, and the other bars decompose the runtime into the main steps of the SmartEM pipeline. For the initial scan and rescan steps, the purple bar represents the lower bound computed by multiplying dwell time by number of pixels. Note that the total time for “mixed-prec., par.” is less than the sum of its components due to parallelization

The timing results under three acquisition settings are shown in Fig. 5. In the first setting, autocasting accelerated the process of computing the rescan map by 43%. Parallelization sped up the total acquisition time by 224 seconds, equivalent to 76% of total computation time. Latency hiding was limited to 76% due to the increased number stage movements under parallelization. Smaller tile sizes in the second setting caused stage movements to comprise a greater fraction of overall runtime, diminishing the advantage of parallelization. Further, mixed-precision inference showed less of an advantage at these smaller tile sizes. The third setting used the same tile sizes and number of tiles per sections as the first setting, so the relative runtime results were similar, with mixed-precision, parallel smart imaging being the fastest acquisition method.

## Conclusion

In this work, we constructed a general model of image acquisition runtime, and showed that smart imaging is faster than traditional imaging in a variety of settings, especially if imaging and computation are parallelized. We demonstrated two examples of how the end-to-end runtime of SmartEM can be modified: using mixed-precision inference to decrease computation time, and parallelizing microscope and support computer operations. When tile sizes were sufficiently large, both modifications decreased the total SmartEM acquisition time. With small tile sizes, tile transition time (e.g. stage movement) is a disproportionately larger part of overall runtime, so the advantage of parallelization disappears (Figs. 2 and 5).

The primary limitation of our theoretical model is the assumption that the user has access to a function that maps rescan ratios to quality values. First, the user needs to be able to quantify image “quality.” Quality might be defined as signal-to-noise ratio, or object detection accuracy. Then, since the function is likely unknown, it has to be estimated from, for example, a random subset of the data. We provide an example of this process in Fig. 4. We note that there is an implicit assumption that the predictor that computes rescan regions performs consistently across the image. We consider predictor performance to be a computer vision topic outside the scope of this work.

Also, we note that our timing equation for the parallelized algorithm does not include bottlenecks that might limit parallelization. In the typical SmartEM setup, the support computer has a dedicated GPU that is used exclusively to compute rescan masks, so it is reasonable to assume there is no resource contention bottleneck with the GPU. However, one might be concerned about synchronization bottlenecks involved in transferring fast dwell time images to the computation thread. In this work, there are only two threads that access shared queues (the imaging thread and computation thread), so synchronization issues should not be as severe as more highly parallelized programs. Further, our empirical experiments suggest that the parallelization speedup is roughly equal to the computation time minus the additional stage movement time, so there does not seem to be a significant synchronization bottleneck in our setup. In order to modify the equations to capture possible bottlenecks that limit parallelization, one could introduce new parameters $T_f'$ and $T_c'$ that incorporate synchronization bottlenecks for the fast scan and computation steps, respectively.

Finally, rather than wanting to achieve a quality standard in minimal time, the user might want to maximize quality in a fixed amount of time (Meirovitch et al. 2024). The equations in our model can be modified to characterize this approach.

In the first and third acquisition settings, mixed-precision inference and parallelization both decreased the overall SmartEM runtime. However, speedup was limited since microscope operations took significantly longer than neural network inference. The advantage of parallelization would be more significant if the latencies of imaging and computation were more closely matched. One of the bottlenecks of imaging is the overhead costs associated with loading and executing rescans, as shown by the discrepancy between the theoretical and measured rescan times in Fig. 5. Rescanning in our experiments was performed with “bitmap” patterns, however there are other patterning techniques available in EM control software such as “stream” patterning. Previous work has shown that, when acquiring sparse images, scan path can affect runtime (Boughorbel et al. 2017). However, we leave this as a potential area of future work.

While improved software can continue to close the gap between theoretical and observed rescan times, there will likely continue to be a gap due to hardware limitations. For example, discontinuous rescan patterns force the electron beam to jump between different regions of the field of view. While beam deflection is fast, it is an example of an overhead cost that keeps rescan time above the simple formula of number of pixels times dwell time per pixel.

This work included the first measurements of end-to-end runtime of a smart imaging pipeline. Our timing model equations generalize to any imaging workflow where there is a tradeoff between imaging time, and image quality. Smart microscopy has so far been limited to EM connectomics, but we believe it also offers significant time saving potential in other fields.

## Data Availability

The code associated with our timing analysis is freely available at https://github.com/Shavit-Lab/timing-apps. SmartEM pipeline software is available upon request.

## Abbreviations

EM: Electron microscopy
SmartEM: Smart electron microscopy
